# Paxillin and Focal Adhesion Kinase (FAK) Regulate Cardiac Contractility in the Zebrafish Heart

**DOI:** 10.1371/journal.pone.0150323

**Published:** 2016-03-08

**Authors:** Sofia Hirth, Anja Bühler, John B. Bührdel, Steven Rudeck, Tillman Dahme, Wolfgang Rottbauer, Steffen Just

**Affiliations:** 1 Molecular Cardiology, University of Ulm, Ulm, Germany; 2 Department of Medicine II, University of Ulm, Ulm, Germany; Medical University Hamburg, University Heart Center, GERMANY

## Abstract

An orchestrated interplay of adaptor and signaling proteins at mechano-sensitive sites is essential to maintain cardiac contractility and when defective leads to heart failure. We recently showed that Integrin-linked Kinase (ILK), ß-Parvin and PINCH form the IPP-complex to grant tuned Protein Kinase B (PKB) signaling in the heart. Loss of one of the IPP-complex components results in destabilization of the whole complex, defective PKB signaling and finally heart failure. Two components of IPP, ILK and ß-Parvin directly bind to Paxillin; however, the impact of this direct interaction on the maintenance of heart function is not known yet. Here, we show that targeted gene inactivation of Paxillin results in progressive decrease of cardiac contractility and heart failure in zebrafish without affecting IPP-complex stability and PKB phosphorylation. However, we found that Paxillin deficiency leads to the destabilization of its known binding partner Focal Adhesion Kinase (FAK) and *vice versa* resulting in degradation of Vinculin and thereby heart failure. Our findings highlight an essential role of Paxillin and FAK in controlling cardiac contractility via the recruitment of Vinculin to mechano-sensitive sites in cardiomyocytes.

## Introduction

Integrin-linked Kinase (ILK) is a crucial regulator of cardiomyocyte contractility [1, 2]. Together with Parvin and PINCH, it forms the ternary IPP-(ILK-Parvin-PINCH) complex which modulates the expression of stretch-responsive genes such as *atrial natriuretic factor* (*anf)* or *vascular endothelial growth factor* (*vegf)* by the regulation of Protein Kinase B (PKB) phosphorylation and activation in the heart [1, 3]. We and others depicted that loss of each of the IPP-complex components leads to IPP-complex destabilization, reduced PKB phosphorylation and finally heart failure in zebrafish, mice and humans [1, 3–7].

Within cardiomyocytes, the IPP-complex is located at focal adhesions and Z-discs where it interacts via ILK and Parvin with Paxillin [8, 9]. Paxillin is a focal adhesion associated adaptor protein that recruits diverse signaling proteins such as Focal adhesion Kinase (FAK) and Vinculin to focal adhesions to guarantee regular signal reception and transduction [10]. In this context, binding between Paxillin and FAK has been described to be important to transmit integrin-dependent signals [11]. Furthermore, Paxillin is known to be important to recruit Vinculin, a key structural component in cardiomyocytes that plays an essential role in mechano-transduction, to focal adhesions [10, 12, 13]. Knock-out studies of Paxillin in mice resulted in early embryonic lethal phenotype including severe developmental cardiac defects [14]. Nevertheless, the role of Paxillin in the vertebrate heart, in particular in regard to its role in orchestration of cardiac contractility, is not yet elucidated.

To decipher for the first time the *in vivo* role of Paxillin in the vertebrate heart, we inactivated Paxillin in the model system zebrafish and observed severely impaired cardiac contractility in Paxillin morphants. We showed that loss of Paxillin does neither impact on IPP-complex stability nor PKB signaling. However, we further demonstrate here that Paxillin-mediated heart failure is caused by the destabilization of the interaction partners Paxillin and FAK, leading to impaired recruitment and subsequent degradation of Vinculin.

## Materials and Methods

### Zebrafish strains and injection procedures

The present study was performed after appropriate institutional approvals, which conform to EU Directive 2010/63/EU. Care and breeding of zebrafish, *Danio rerio*, was conducted as described in [15, 16]. Briefly, the embryos were bred and maintained under standard conditions at 28.5°C. Fertilized eggs and embryos were kept in regular embryo medium E3 (5 mM NaCl, 0.17 mM KCl, 0.33 mM CaCl2 and 0.33 mM MgSO4 dissolved in water). Embryos were staged as described previously in hours post fertilization (hpf) [17]. For documentation, 0.003% 1-phenyl-2-thiourea was added to the embryo medium E3 to delay pigmentation. Morpholino-modified antisense oligonucleotides (MOs; Gene Tools, LLC, Oregon, USA) were directed either against the translational start site and/or splice-acceptor/donor site of zebrafish *paxillin* (MO1-*paxillin*, MO2-*paxillin*), *focal adhesion kinase 1a* (MO1-*fak1a*, MO2-*fak1a)*, *focal adhesion kinase 1b* (MO1-*fak1b*, MO2-*fak1b*) and *vinculin* (MO-*vinculin*). As negative controls standard control MO or five base pair mismatch MOs were injected at the same concentration as the respective MO. The standard control MO was only used for co-immunofluorescence stainings. For all other experiments the corresponding mismatch MO was used. All MOs were resolved in KCl and injections were performed into one-cell zebrafish embryos (for MO sequences, MO concentrations and accession numbers of genes see S1 Table).

For rescue experiments, sense-capped mRNA of *myc*-tagged zebrafish and human *paxillin* was synthesized using the mMESSAGE mMACHINE system (Ambion, Cat.No. AM1340). 1 ng of zebrafish *paxillin* mRNA and 3 ng of human *paxillin* mRNA was injected.

### RNA extraction and quantitative real-time PCR

For RNA extraction 25 embryos were collected at 72 hpf for each sample. To extract the RNA the RNeasy^®^ Mini Kit was used (Qiagen) according to the manufacturer’s instructions. Reverse transcription was performed by using SuperScript^®^ III Reverse Transcriptase (Life Technologies).

Quantitative real-time PCR was carried out according to standard protocols using SYBR-Green master mix (Roche) and a Roche LightCycler 480 II. cDNA was generated as described above from 72 hpf old embryos (for primer sequences see S2 Table). To correct sample to sample variation house-keeping genes *rpl13a* and β-*actin2* were used for normalization (for primer sequences see S2 Table).

For splice detection assays RNA was isolated from 50 embryos at 24 hpf using Qiazol Lysis reagent (Qiagen) subsequent RT-PCR was performed with primers for the specific knock-down (for primer sequences see S2 Table).

### Primary cell culture of adult zebrafish cardiomyocytes and immunofluorescence staining of adult zebrafish cardiomyocytes and embryonic hearts

Extraction of adult zebrafish hearts for preparation of primary cell cultures was approved by the local Animal Ethics Committee (Tierforschungszentrum Ulm, protocol number o.183) in accordance to governmental and international guidelines on animal experiments. Cardiomyocytes of adult zebrafish were isolated and cultured as described previously [18]. Briefly, hearts of adult zebrafish (<6 month) were isolated and ventricles were digested with collagenase for 2 h at 32°C. Afterwards cardiomyocytes were cultured on fibronectin-coated 8 well cover slides at 28°C with 5% CO_2_ for up to 4 weeks. Per well 4 adult hearts were used. For immunofluorescence staining, cardiomyocytes were fixed in 4% methanol-free formaldehyde in PBS, permeabilized in 0.05% Triton-X-100 and blocked in 0.5% BSA in PBST. As primary antibodies, polyclonal rabbit anti-human FAK-C20 (1:100; Santa Cruz#sc558), mouse anti-chicken Paxillin (1:100; BD Transduction Laboratories #610051), monoclonal mouse anti-α-actinin (1:200; Sigma Aldrich #A5044) and rabbit anti-sarcomeric actinin (1:100; abcam #ab109776) were used. As secondary antibodies, goat anti-mouse IgG1 Alexa Fluor 488 or 594 and goat anti-rabbit IgG Alexa Fluor 488 or 594 (1:1000; Thermo Fisher Scientific) were used.

Embryonic zebrafish hearts were isolated and stained as described previously in [19]. Briefly, dissected hearts of 72 hpf old embryos were fixed in 4% methanol-free formaldehyde in PBS. After blocking in 10% goat-serum in PBST, hearts were incubated with the primary antibodies, anti-vinculin (1:400; abcam #ab91459) and anti-β-catenin (1:400; abcam #ab6301) for 1 h at RT. The secondary antibodies were diluted 1:1000 in PBST and incubated for 30 min at RT. For documentation a Zeiss Axioskop2 plus and the Axio Vision software (Zeiss) was used.

### Isolation of zebrafish protein lysates, western blot analysis and *in vitro* synthesis of proteins

For the preparation of zebrafish protein lysates, 50 embryos of each group were collected after 72 hpf and deyolked. Then they were washed and homogenized with the help of a pestle. Afterwards, samples were incubated for 15 min on ice and then centrifuged for 10 min at 4°C. Supernatant was collected and protein concentration was determined by a Bradford protein assay.

For western blot analysis 20 μg of each protein lysate was boiled in 3x Laemmli Buffer and loaded on a precast 10% SDS gel (Bio-Rad). Proteins were separated by SDS-PAGE and transferred to polyvinylidene fluoride (PVDF) membrane. After blocking in 5% milk powder in TBST or 5% BSA (bovine serum albumin) in TBST, depending on the used primary antibody, for 2h at RT, the membrane was incubated with the primary antibodies over night at 4°C. The secondary antibodies were incubated for 2h at RT.

The following primary antibodies were used: polyclonal rabbit anti-human FAK-C20 antibody (1:1000, Santa Cruz; #sc558), mouse anti-chicken Paxillin (1:1000; BD Transduction Laboratories #610051), rabbit anti-zebrafish ILK (1:500; SAB2701733), monoclonal mouse anti-LIMS1 [PINCH-C58] (1:1000; abcam #ab50305) polyclonal rabbit anti-AKT1/2/3 (1:1000; Santa Cruz #8312), polyclonal rabbit anti-phospho-AKT (Ser497) (1:1000; Cell Signaling #9271) and polyclonal rabbit anti-Vinculin (1:1000; abcam #ab91459). Loading controls included mouse anti-β-actin (1:1000; Sigma-Aldrich #A5441) and rabbit anti-pan cadherin (1:50000; Abcam #ab16505). Signals were detected by chemiluminescence (anti-mouse IgG HRP-linked, anti-rabbit IgG HRP-linked, Cell signaling #7076/#7074) using a luminescent image analyzer (Image Quant Las4000 mini).

*In-vitro* synthesized Paxillin protein was generated following standard protocols (TnT Kit, Promega) and used as positive control for western blot analysis.

### Functional assessment and statistical analysis

Still images were taken with an Olympus SZX 16 microscope and movies were recorded with a Leica DM IL LED microscope. The functional assessment of cardiac contractility was carried out as described [16, 20]. Fractional shortening was determined by measuring the diameters of the ventricle at the end of contraction (systole) and relaxation (diastole) diameters using the zebraFS software (http://www.benegfx.de), then FS was calculated using the following formula:

$$\text{FS}\;=\;\frac{\emptyset \text{diastole}-\emptyset \text{systole}}{\emptyset \text{diastole}}.$$

All results are expressed as mean ± s.d. and analysis were performed using unpaired Student's *t*-test, a value of *P*<0.05 was accepted as statistically significant.

## Results

### Paxillin is essential to maintain cardiac function in zebrafish

**Fig 1:**
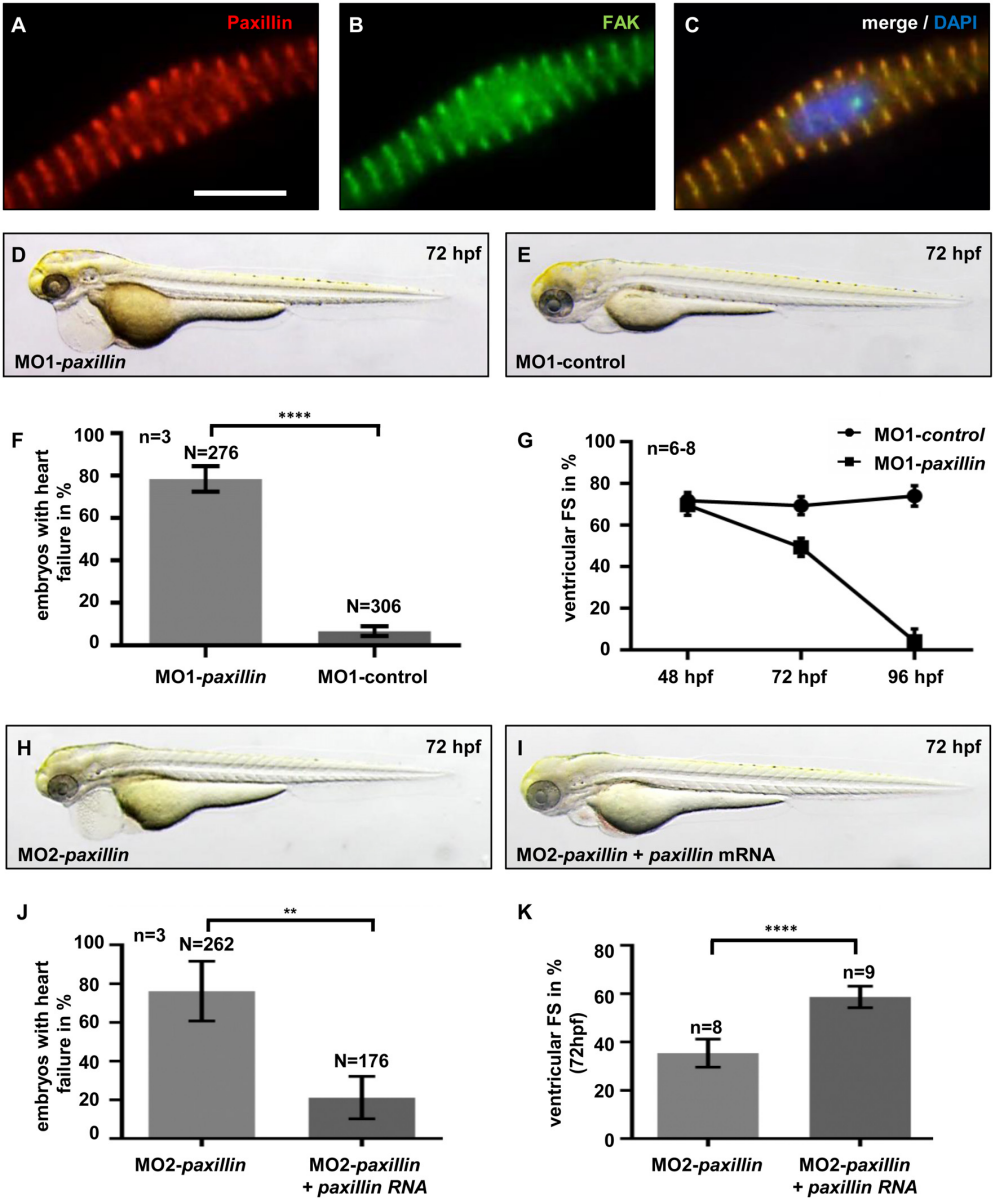
Targeted knock-down of Paxillin leads to heart failure in zebrafish. **(A-C)** Co-immunostaining of Paxillin (A, red) and the known Z-disk protein Focal Adhesion Kinase (FAK) (B, green) on adult primary zebrafish cardiomyocytes revealed localization of Paxillin to sarcomeric Z-disks. Cell nuclei were counterstained with DAPI (C, blue). Scale bar 10 μm. **(D-G)** Lateral view of *paxillin* start MO (MO1-*paxillin*) (D) and *paxillin* 5bp-mismatch-MO (MO1-control) (E) injected embryos at 72 hpf. (F) 78% of embryos injected with 5.4 ng MO1-*paxillin* developed pericardial edema and blood congestion at the cardiac inflow tract (n = 3; *P<0.0001), whereas control-injected embryos developed no pathological phenotype. (G) Fractional shortening (FS) measurements of Paxillin morphant ventricles at 48, 72 and 96 hpf. FS of Paxillin morphant ventricles was slightly reduced to 69.6% ± 5.07% compared to corresponding 5bp-mismatch-MO injected embryos (FS: 71.6% ± 3.96%) at 48 hpf. At 72 hpf, FS in Paxillin morphants was reduced to 49.29% ± 4.39% compared to control morphants (FS: 69.3% ± 4.36%), whereas ventricular chambers of Paxillin morphants became almost silent (FS: 4.23% ± 6.17%) compared to controls (FS: 74% ± 4.96%) at 96 hpf (n = 6–8 individuals per time point). **(H-K)** Lateral view of MO2-*paxillin* (H) and MO2-*paxillin*+*paxillin* mRNA (I) injected embryos at 72 hpf. (J) Co-injection of 4.5 ng of MO2-*paxillin* and 1 ng wild-type *paxillin* mRNA rescued the Paxillin morphant heart failure phenotype (n = 3; *P = 0.0073). (K) Quantification of ventricular FS revealed that ectopic expression of *paxillin* mRNA significantly improved contractile function in Paxillin morphants at 72 hpf (n = 8–9 individuals; *P<0.0001). Error bars indicate s.d.

To study for the first time the *in vivo* role of Paxillin in the vertebrate heart, we isolated the zebrafish orthologous sequence by BLAST analysis. Zebrafish Paxillin (GenBank: NM_201588.1) displays high amino acid identity compared to human and murine Paxillin (GenBank: U14588.1 and NP_035353.1), especially in the evolutionary conserved motifs and domains that are known to mediate protein-protein interactions, such as LD motifs, LIM domains, SH2 domain-binding sites and an SH3 domain-binding site (S1 Fig). By immunostaining, we revealed that next to its structure its expression pattern in cardiomyocytes is also conserved between mammals and zebrafish with strong expression of Paxillin at the sarcomeric Z-disk and the sarcolemma (Fig 1A–1C).

To elucidate the impact of Paxillin on vertebrate heart function, we inactivated zebrafish Paxillin by Morpholino-modified antisense oligonucleotide (MO) mediated gene knock-down using either a MO directed against the translational start site of *paxillin* (MO1-*paxillin*) or one targeting the splice-acceptor site of exon 2 (MO2-*paxillin*). As shown in Fig 1D–1F, by 72 hpf, 78.46% ± 6.05% of MO1-*paxillin* injected embryos developed progressive heart failure characterized by reduced ventricular systolic force, severely reduced blood flow, blood congestion at the cardiac inflow tract and pericardial edema (S1 and S2 Movies). Fractional shortening (FS) of *paxillin* morphant ventricles was reduced to 49.29% ± 4.39% compared to control morphants (FS: 69.3% ± 4.36%) at 72 hpf. By 96 hpf, the ventricular chambers of *paxillin* morphants became almost silent (FS: MO1-*paxillin* 4.23% ± 6.17% vs. MO1-control 74% ± 4.9%) (Fig 1G). MO1-*paxillin* injected embryos die five days post fertilization due to their contractile dysfunction.

To investigate the efficacy of MO1-*paxillin*, we assessed Paxillin protein levels in morphant embryos and found that injection of MO1-*paxillin* resulted in severely reduced protein levels of Paxillin (S2J Fig). To validate that the observed heart failure phenotype of *paxillin* morphants is specific, we performed rescue experiments. Therefore, we co-injected 3 ng of human *paxillin* mRNA together with 5.4 ng MO1-*paxillin*. We found that ectopic expression of human *paxillin* mRNA was able to significantly improve contractile force in MO1-*paxillin* morphants (S2A–S2D Fig). Only 17.2% ± 6.8% of co-injected embryos developed a heart failure phenotype, whereas 80.32% ± 14.4% of MO1-*paxillin* injected embryos were affected (S2C Fig). Fractional shortening measurements at 72 hpf revealed that contractile force of embryos co-injected with human *paxillin* mRNA was significantly improved to 61.58% ± 3.4% compared to MO1-*paxillin* injected embryos which display a FS of 39.44% ± 6.2% (S2D Fig). Moreover, injection of a second, independent splice site targeting MO (MO2-*paxillin*) led to a similar heart failure phenotype as injection of MO1-*paxillin*, 82.27% ± 7.9% of MO2-*paxillin* injected embryos developed contractile dysfunction, whereas only 9.5% ± 2.6% of control-injected embryos showed a comparable phenotype (S2E–S2G Fig). Determination of ventricular FS showed that contractile force was reduced to 46.5% ± 5.2% compared to control morphants (FS: 69.24% ± 3.8%) at 72 hpf. By 96 hpf, Paxillin morphant ventricles were almost silent (FS: MO2-*paxillin* 3.06% ± 5.78% vs. MO2-control 72.84% ± 5.25%) (S2H Fig). Isolation of mRNA from MO2-*paxillin* injected embryos and subsequent RT-PCR confirmed the predicted effect on mRNA splicing (S2I Fig). Furthermore, ectopic expression of 1 ng wild-type zebrafish *paxillin* mRNA rescued ventricular contractile function in *paxillin* morphant embryos (Fig 1H–1K). Whereas 76.1% ± 15.4% of MO2-*paxillin* injected embryos developed contractile dysfunction, only 21.1% ± 10.95% of the embryos injected with MO2-*paxillin* and *paxillin* mRNA exhibited reduced cardiac contractility and heart failure (Fig 1J). FS measurements revealed a significant improvement of contractile force from 35.45% ± 5.5% (MO2-*paxillin*) to 58.6% ± 4.7% in rescued embryos (MO2-*paxillin*+mRNA) at 72 hpf (Fig 1K). These findings indicate that the effects on cardiac function induced by MOs targeting Paxillin were specific and not due to off-target effects.

### Paxillin deficiency does not interfere with IPP-PKB signaling

**Fig 2:**
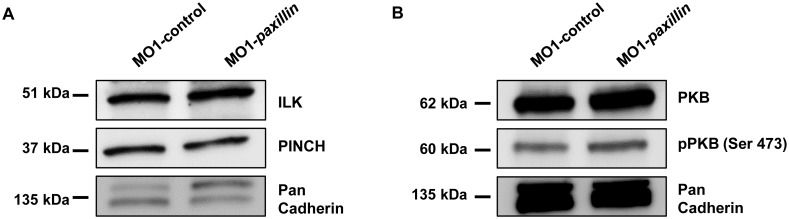
Paxillin does not regulate IPP-complex stability and PKB activation in zebrafish. **(A)** Western blot analysis of Paxillin morphant protein lysates compared to lysates obtained from control-injected embryos with antibodies against ILK and PINCH. For each sample 50 embryos were pooled and 20 μg of protein lysate were loaded per lane. The figure shows one representative western blot out of three independent experiments. **(B)** Western blot analysis of protein levels of total PKB and serine 473-phosphorylated PKB in MO1-*paxillin*-injected embryos. For each sample 50 embryos were pooled and 20 μg of protein lysate were loaded per lane. The figure shows one representative western blot out of three independent experiments.

The ILK-Parvin-PINCH- (IPP-) complex regulates contractility by granting phosphorylation and activation of PKB/AKT in cardiomyocytes. Loss of one of the IPP-complex components results in destabilization of the whole complex, defective PKB signaling and finally heart failure [1, 3]. First, to investigate if loss of the IPP-complex interaction partner Paxillin also impacts IPP-complex stability, we evaluated protein levels of the IPP-complex components ILK and PINCH in Paxillin-deficient embryos. As revealed by western blot analyses, protein levels of ILK or PINCH were not diminished in Paxillin morphant embryos (Fig 2A), indicating that loss of Paxillin did not impact IPP-complex stability *in vivo*. Accordingly, PKB phosphorylation at Serine 473 downstream of the IPP-complex was not reduced in Paxillin morphants (Fig 2B), further indicating that the effect of Paxillin on systolic cardiac function was not mediated via altered IPP-PKB signaling.

### Loss of Focal Adhesion Kinase (FAK) leads to heart failure and decreased Paxillin protein levels in zebrafish embryos

**Fig 3:**
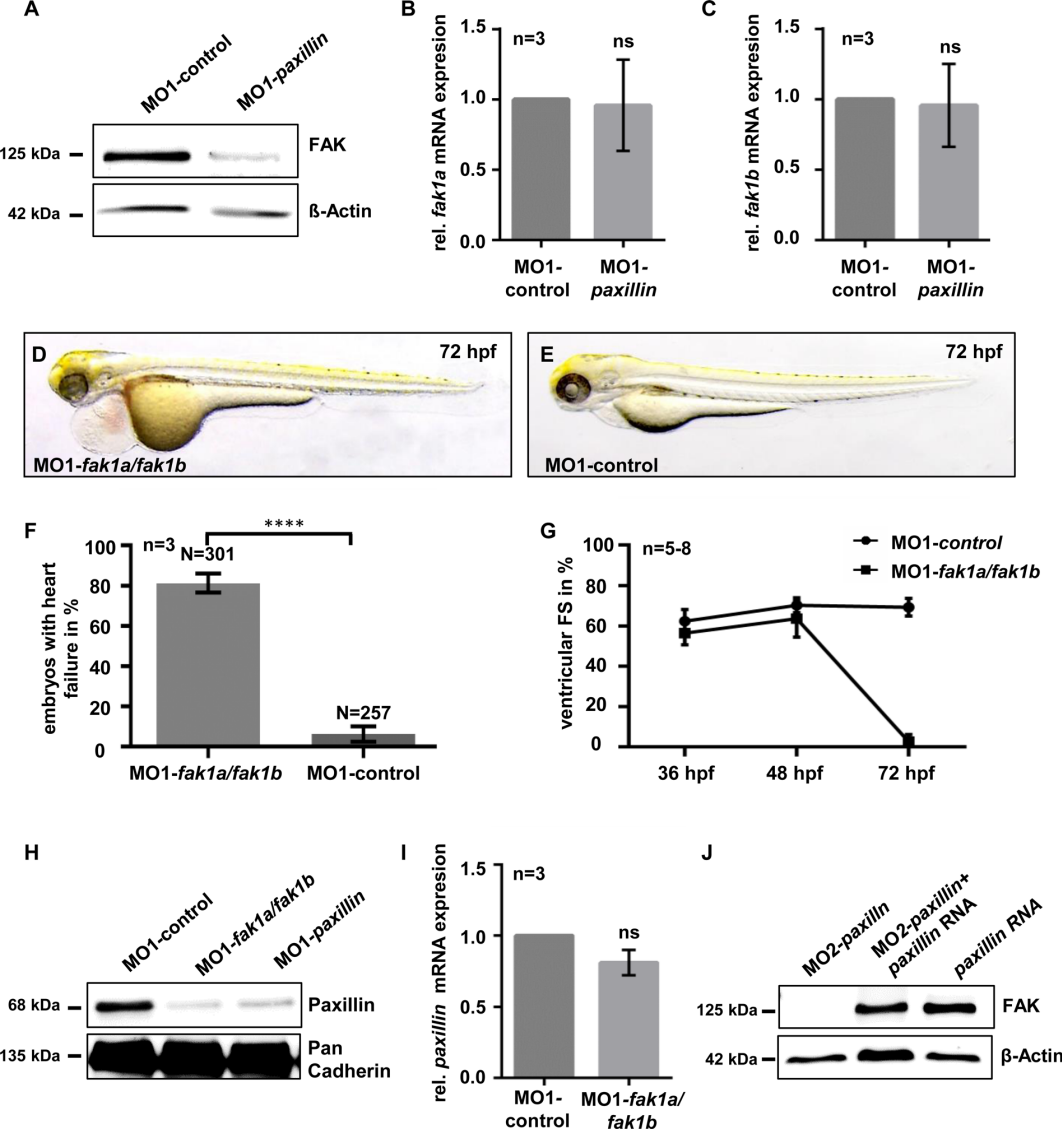
Knock-down of *fak1a* and *fak1b* phenocopies the Paxillin morphant phenotype. **(A)** Western blot analysis of FAK protein levels in Paxillin-deficient zebrafish embryos compared to control-injected embryos. For each sample 50 embryos were pooled and 20 μg of protein lysate were loaded per lane. The figure shows one representative western blot out of three independent experiments. **(B, C)** Quantitative real time PCR of Paxillin-depleted and control-injected embryos showed no significant (ns) alteration of *fak1a* (n = 3; *P = 0.9017) (B) and *fak1b* (n = 3; *P = 0.8878) (C) mRNA expression. For statistical analysis student’s *t*-test was performed. **(D-F)** Lateral view of MO1-*fak1a*/*fak1b* (D) and 5bp-mismatch-MOs (MO1-control) (E) injected embryos at 72 hpf. Embryos co-injected with 2.7 ng of MO1-*fak1a* and 2.15 ng of MO1-*fak1b* showed a severe heart failure phenotype whereas injection of the same amount of *fak1a* and *fak1b* 5bp-mismatch MOs (MO1-control) caused no significant pathological cardiac phenotype (F) (n = 3; *P<0.0001). **(G)** FS measurements of FAK morphant ventricles at 36, 48 and 72 hpf. FS of FAK double-knock-down morphant ventricles was slightly reduced to 56.38% ± 5.85% compared to corresponding 5bp-mismatch-MO injected embryos (FS: 62.4% ± 5.77%) at 36 hpf. At 48 hpf, FS in FAK morphants was reduced to 63.57% ± 9.18% compared to control morphants (70.33% ± 3.72%), whereas ventricular chambers of FAK-deficient embryos became almost silent (FS: 2.6% ± 3.71%) compared to controls (FS: 69.33% ± 4.36%) at 72 hpf (n = 5–8 individuals per time point). **(H)** Western blot analysis of Paxillin levels in control-, MO1-*fak1a*/*fak1b-* and MO1-*paxillin*-injected embryos. Paxillin protein levels are severely reduced after targeted knock-down of FAK and Paxillin, respectively. For each sample 50 embryos were pooled and 20 μg of protein lysate were loaded per lane. The figure shows one representative western blot out of three independent experiments. **(I)** Quantitative real time PCR of FAK-depleted and control-injected embryos showed no significant (ns) alteration of *paxillin* mRNA expression (n = 3; *P = 0.0937). For statistical analysis student’s *t*-test was performed. **(J)** Western blot analysis of embryos co-injected with zebrafish *paxillin* mRNA and MO2-*paxillin* compared with embryos injected with MO2-*paxillin* or *paxillin* mRNA alone. Ectopic expression of zebrafish *paxillin* mRNA was able to restore FAK protein expression. For each sample 50 embryos were pooled and 20 μg of protein lysate were loaded per lane. Error bars indicate s.d.

To evaluate the mechanism by which Paxillin controls cardiac contractility, we assayed Focal Adhesion Kinase (FAK) protein levels, since it is known that Paxillin not only interacts with proteins of the IPP-complex but also binds to FAK [21]. Interestingly, FAK protein levels were severely reduced in Paxillin morphants (Fig 3A), suggesting that Paxillin is essential to warrant FAK protein stability *in vivo*. Remarkably, western blot analysis revealed that ectopic expression of zebrafish *paxillin* mRNA in Paxillin morphants was able to restore FAK protein expression (Fig 3I). By quantitative real time PCR, we assessed that mRNA levels of neither *fak1a* nor *fak1b* were affected in Paxillin-deficient embryos (Fig 3B and 3C), demonstrating that loss of Paxillin had no impact on *fak1a/fak1b* transcription.

Since Paxillin associated heart failure seems to be mediated via FAK degradation on protein level, we inactivated FAK in zebrafish by gene knock-down. Therefore, we simultaneously targeted both zebrafish FAK orthologs (AF348085.1 and AY196213.1) by MOs directed against the respective translational start sites (MO1-*fak1a* and MO1-*fak1b*). At 72 hpf, 81.3% ± 4.6% of embryos co-injected with MO1-*fak1a* and MO1-*fak1b* developed heart failure similar to Paxillin-deficient zebrafish embryos. This phenotype was accompanied by progressive reduction of ventricular contractility, blood congestion at the sinus venosus and a pronounced pericardial edema (Fig 3D–3G; S3 Movie). Fractional shortening (FS) of *fak1a/fak1b* morphant ventricular chambers was reduced to 63.57% ± 9.18% compared to corresponding 5bp-mismatch-MO injected embryos (FS: 70.33% ± 3.72%) at 48 hpf and to 2.6% ± 3.71% compared to control morphants (FS: 69.33% ± 4.36%) at 72 hpf (Fig 3G).

By western blot analysis, we found that the amount of FAK proteins was severely decreased in *fak1a/fak1b* morphants compared to controls (S3D Fig). Co-injection of two additional, independent splice site targeting MOs, MO2-*fak1a* and MO2-*fak1b*, respectively, led to a similar heart failure phenotype as observed after co-injection of MO1-*fak1a* and MO1-*fak1b*, whereas co-injection of the respective mismatch MOs caused no cardiac phenotype (S3A and S3B Fig). By RT-PCR, we confirmed effective blocking of *fak1a* and *fak1b* mRNA processing, predicted to cause premature termination of Fak1a and Fak1b protein translation (S3C Fig).

To test whether FAK is also crucial for Paxillin stability *in vivo*, we next evaluated Paxillin protein levels in FAK-deficient zebrafish and found indeed severely reduced Paxillin levels (Fig 3H). By quantitative real-time PCR, we showed that mRNA expression of *paxillin* in FAK-depleted embryos was not altered compared to control-injected embryos (Fig 3I). Remarkably, western blot analysis revealed that ectopic expression of zebrafish *paxillin* mRNA in Paxillin morphants was able to restore FAK protein levels (Fig 3J). In summary, these data indicate that interaction of Paxillin and FAK might be essential to warrant proper heart function in zebrafish embryos.

### Vinculin is down-regulated in Paxillin- and FAK-depleted zebrafish embryos

**Fig 4:**
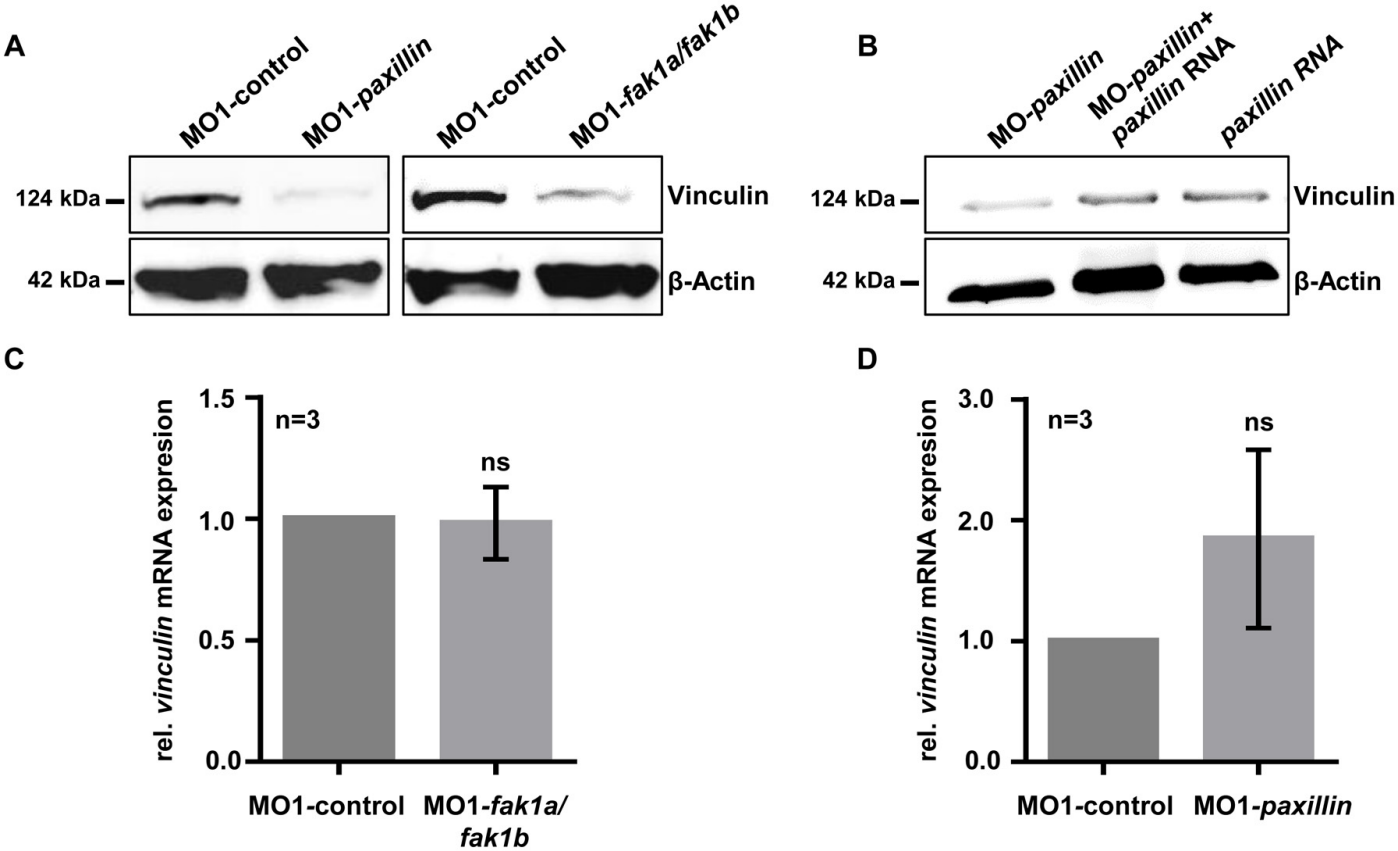
Vinculin protein levels are severely decreased in Paxillin- and FAK-deficient zebrafish embryos. **(A)** Western blot analysis of MO-mediated Paxillin inactivation (MO1-*paxillin)* led to severely reduced Vinculin protein levels *in vivo*. Similar to loss of Paxillin, targeted ablation of FAK (MO1-*fak1a*/*fak1b)* also resulted in degradation of Vinculin, whereas Vinculin levels in control-injected embryos were completely unaffected. For each sample 50 embryos were pooled and 20 μg of protein lysate were loaded per lane. The figure shows one representative western blot out of three independent experiments. **(B)** Western blot analysis of embryos co-injected with zebrafish *paxillin* mRNA and MO2-*paxillin* compared with embryos injected with MO-*paxillin* or *paxillin* mRNA alone. Ectopic expression of zebrafish *paxillin* mRNA was able to restore Vinculin protein levels. For each sample 50 embryos were pooled and 20 μg of protein lysate were loaded per lane. **(C, D)** Bar graphs compare average of *vinculin* mRNA expression in Paxillin- (n = 3; *P = 0.1179) (C) and FAK-depleted (n = 3; *P = 0.8427) (D) compared to control-injected embryos. For statistical analysis student’s *t*-test was performed.

To further decipher the molecular mechanism by which Paxillin- and FAK deficiency led to contractile dysfunction and heart failure in the zebrafish heart *in vivo*, we investigated the role of Vinculin in Paxillin/FAK-mediated heart failure, since it was recently shown that FAK and Paxillin are required to recruit Vinculin to Focal Adhesion (FA) sites, at least in mouse embryonic fibroblasts [22]. Hence, to evaluate whether the interplay between Paxillin and FAK is crucial to regulate cardiac contractile force via Vinculin recruitment, we determined Vinculin protein levels in FAK- and Paxillin-depleted zebrafish embryos by western blot analysis. Remarkably, Vinculin protein levels were severely down-regulated in both, FAK- and Paxillin-depleted zebrafish (Fig 4A). Vinculin protein expression was completely restored by the ectopic expression of zebrafish *paxillin* mRNA in MO2-*paxillin* injected embryos (Fig 4B). By quantitative real-time PCR, we showed that neither loss of Paxillin nor FAK affected *vinculin* mRNA transcription, suggesting that indeed Vinculin proteins were degraded. (Fig 4C and 4D).

**Fig 5:**
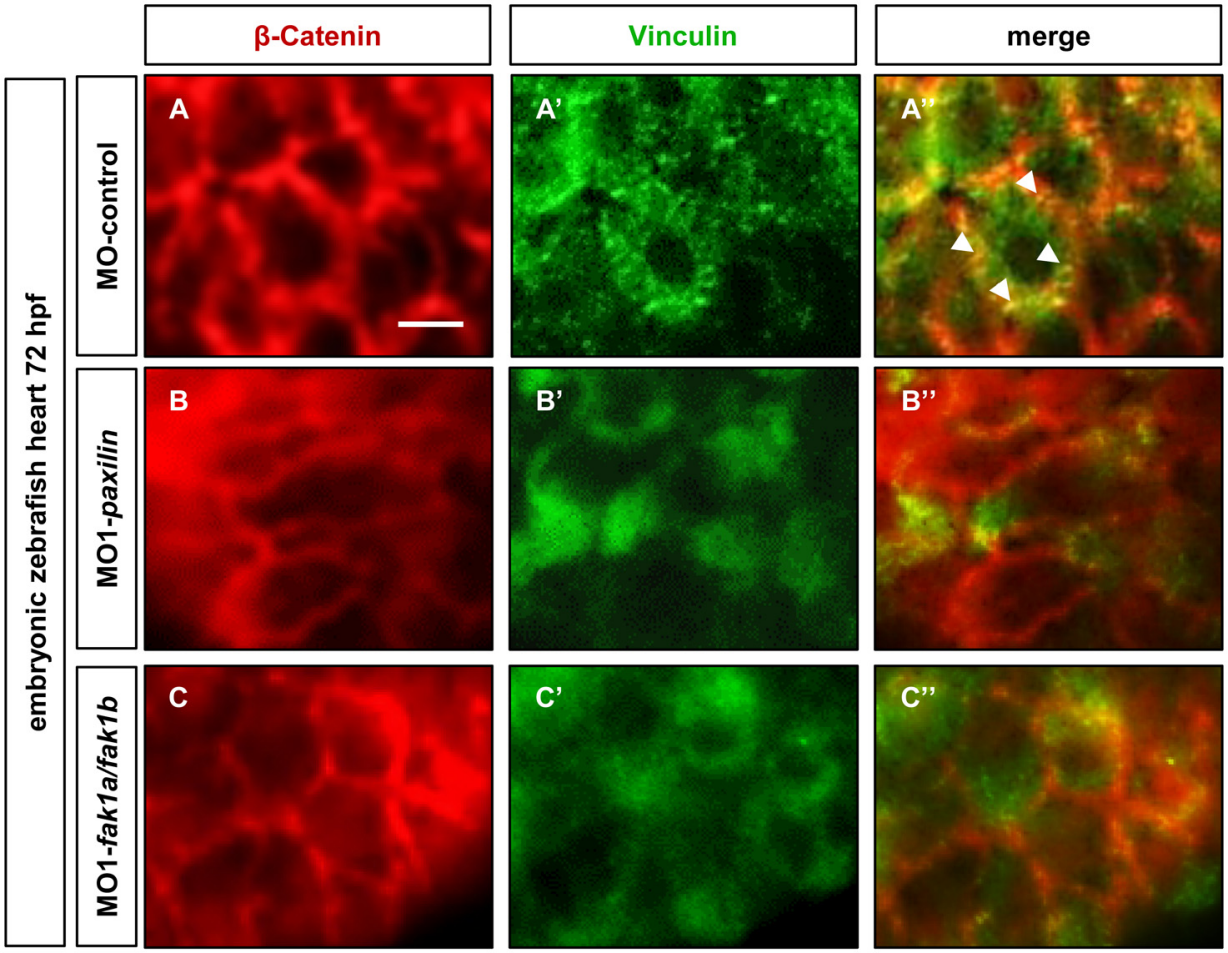
Vinculin does not localize to focal adhesion sites in Paxillin- and FAK-deficient zebrafish embryos. **(A-C)** Co-immunostaining of dissected embryonic (72 hpf) zebrafish hearts with antibodies against β-Catenin (red) and Vinculin (green) of control- (A), MO1-*fak1a/fak1b-* (B) and MO1-*paxillin*- (C) injected hearts. Arrow heads indicate focal adhesion sites. Scale bar 5μM.

Moreover, we performed co-immunostaining with antibodies against β-Catenin and Vinculin on hearts dissected from FAK- and Paxillin-depleted as well as control-injected embryos at 72 hpf. Whereas in control morphant hearts Vinculin properly localized to focal adhesion sites, Vinculin was mislocalized and seems to get degraded in morphant hearts (Fig 5A–5C). Finally, we found that targeted gene knock-down of *vinculin*, similar to Paxillin and FAK ablation, results in severe ventricular contractile dysfunction in zebrafish embryos [5] (S4A–S4D Fig).

In summary, these findings imply that both, Paxillin and FAK, seem to be essential to recruit and stabilize Vinculin at focal adhesion sites and that an orchestrated interplay of these three proteins might be essential to maintain contractility in the zebrafish heart *in vivo*.

## Discussion

We show here for the first time that an orchestrated interplay of Paxillin, Focal Adhesion Kinase (FAK) and Vinculin localizing to sarcomeric Z-disks and costameres in cardiomyocytes is essential for the regulation of cardiac contractility. We found that heart failure in Paxillin-deficient zebrafish embryos was caused by the degradation of these interacting proteins. Furthermore, we show here that loss of Paxillin does neither impact on the stability of the Integrin-linked Kinase (ILK)-Parvin-PINCH (IPP)-complex nor on IPP-mediated Protein Kinase B (PKB) activation.

Targeted knock-out of *paxillin* in mice leads to early embryonic lethality around day E9.5 due to abnormal development of mesodermal derived organs such as the heart and somites [14], hindering the analysis of the role of Paxillin in the regulation of cardiac contractility *in vivo*. We demonstrate here that loss of *paxillin* in zebrafish led to severe heart failure, characterized by reduced cardiac contractility. Paxillin is known to interact with the Z-disc-associated IPP-complex. Interestingly, mutations within IPP-complex components lead to destabilization of the complex, reduced phosphorylation of PKB and finally heart failure, similar to Paxillin morphants [1, 3]. However, as shown here, Paxillin seems not to mediate its effect on cardiac function via IPP-signaling, since neither IPP-complex stability nor IPP-dependent PKB activation was altered in Paxillin-associated heart failure.

The multi-adaptor protein Paxillin can also interact at mechanical integration sites with other proteins such as FAK. It was recently determined that Paxillin-FAK binding warrants their activation via the Proto-oncogene tyrosine-protein kinase Src (Src) and hence the recruitment of Vinculin to the costameres. Vinculin is a membrane-cytoskeletal protein that was shown to be important for transmitting mechanical forces and orchestrating mechanical signaling events [22, 23]. Absence of both, FAK and Vinculin is associated with heart failure in mice and humans [5, 13, 24–26]. Furthermore, we recently found that targeted inactivation of Vinculin in zebrafish also leads to severe contractile dysfunction and heart failure *in vivo* [5] (S4A–S4D Fig). As shown here, Paxillin deficiency indeed leads to the degradation of Paxillin and FAK proteins. This results in impaired recruitment and thereby destabilization of Vinculin. *Vice versa*, FAK absence leads to destabilization of Paxillin suggesting that Paxillin and FAK not only interact but also stabilize each other *in vivo* and that this functional interplay is essential for the regulation of cardiac contractility in the zebrafish embryo. Interestingly, in Paxillin deficient mice, FAK protein stability seems not to be severely affected but rather specifically its phosphorylation. However, in mice FAK-Paxillin complex stability might be preserved through up-regulation of the Paxillin orthologue Hic-5 that was shown, similar to Paxillin, to interact with FAK and Vinculin [27, 28]. In zebrafish, no Hic-5 orthologue was identified so far, suggesting that either no genetic compensation at all might be present in Paxillin morphant zebrafish or that the manifestation of heart failure in developing Paxillin-deficient zebrafish is too fast to induce a compensatory HIC-5-mediated cascade.

In fibroblasts, FAK-mediated Paxillin phosphorylation is required to warrant proper recruitment of Vinculin to focal adhesions [22]. As shown here, in cardiomyocytes, targeted ablation of Paxillin also led to the mislocalization of Vinculin, its destabilization and subsequent degradation resulting in heart failure in zebrafish.

In summary, the work reported here implies that Paxillin and FAK are both required to recruit and stabilize Vinculin at sites of force transmission to regulate cardiac contractility in the vertebrate heart. Accordingly, careful analyses of both candidate genes, Paxillin and FAK, might help to further dissect the molecular pathogenesis of human heart failure.

## Supporting Information

S1 Fig Zebrafish Paxillin displays high amino acid sequence homology to murine and human Paxillin. Amino acid sequence alignment of zebrafish (dr), mouse (mm) and human (hs) Paxillin, demonstrating high cross-species homology. Identical amino acids are shaded in black and shown as asterisks in the consensus line. Aminio acids with similar chemicals properties are shaded in gray. Highly homologous motifs such as the LD motifs and LIM domains are indicated.
(PDF)

S2 Fig Injection of human *paxillin* mRNA rescues the cardiac phenotype of MO1-Paxillin morphants. **(A-C)** Lateral view of MO1-*paxillin* (A) and MO1-*paxillin*+human *paxillin* mRNA (B) injected embryos at 72 hpf. (C) Bar graphs compare average of rescued embryos after co-injection of human *paxillin* mRNA compared to MO1-*paxillin* injected embryos (n = 3; *P = 0.0073). **(D)** Fractional shortening (FS) measurement of rescued embryos compared to Paxillin morphant embryos at 72 hpf (n = 5). **(E, F)** Lateral view of a (E) 5bp-mismatch MO (MO2-control) and (F) MO2-*paxillin* splice MO injected embryos at 72 hpf. The heart failure phenotype of MO2-*paxillin* splice morphants was identical to that of embryos injected with translation blocking *paxillin* MO (MO1-*paxillin*). **(G)** Bar graphs compare average of affected embryos after injection of MO2-*paxillin* compared to 5bp-mismatch MO (MO2-control) injected embryos at 72 hpf (n = 3; *P = 0.0001). **(H)** FS measurements of Paxillin morphant ventricles at 48, 72 and 96 hpf (n = 5 individuals per time point). **(I)** RT-PCR of control- and MO2-*paxillin*-injected embryos. Injection of MO2-*paxillin* caused partial and whole skipping of exon 2 (298 bp) leading to premature termination of Paxillin protein translation. Wild-type *paxillin* RNA was severely reduced in Paxillin morphants (527 bp product). **(J)** Western blot analysis of control- (MO1-control) and MO1-*paxillin*-injected embryos demonstrated high efficacy of MO-mediated *paxillin* gene knock-down. For each sample 50 embryos were pooled and 20 μg of protein lysate were loaded per lane. *In vitro* translated (IVT) zebrafish Paxillin protein was used as positive control.
(PDF)

S3 Fig Co-injection of MO2-*fak1a* and MO2-*fak1b* results in defective splicing and heart failure *in vivo*. **(A, B)** Lateral view of (A) control MO (MO2-*control)* and (B) MO2-*fak1a/fak1b*-injected embryos at 72 hpf. The heart failure phenotype of *fak1a*/*fak1b* splice morphants was identical to that of embryos injected with the translation blocking FAK MOs (MO1-*fak1a/fak1b)*. **(C)** RT-PCR of control-, MO2-*fak1a-* and MO2-*fak1b*-injected embryos. Injection of MO2-*fak1a* and MO2-*fak1b* caused intron integration (808 bp MO2-*fak1a*; 883 bp MO2-*fak1b*) leading to premature termination of FAK1a and FAK1b protein translation, respectively. Wild-type *fak1a* and *fak1b* RNA was severely reduced in the respective morphants (150 bp MO2-*fak1a*; 474 bp MO2-*fak1b*). **(D)** Western Blot analysis of control and fak1a/fak1b morphant embryos with an antibody against FAK. For each sample 50 embryos were pooled and 20 μg of protein lysate were loaded per lane.
(PDF)

S4 Fig Knockdown of Vinculin results in cardiac contractile dysfunction. **(A, B)** Lateral view of MO-*vinculin* (A) and *vinculin* 5bp-mismatch-MO (MO-control) (B) injected embryos at 72 hpf. **(C)** Bar graphs compare average of affected embryos after MO injection (MO-*vinculin* 82.85% ± 6.95%; MO-control 7.3% ± 3.76%; *P<0.0001). **(D)** Fractional shortening (FS) measurements of Vinculin morphant ventricles compared to control injected embryos at 48, 72 and 96 hpf. FS of Vinculin morphant ventricles was not affected compared to corresponding 5bp-mismatch-MO injected embryos (MO-*vinculin* 68.01% ± 3.52% vs. MO-control: 66.08% ± 3.12%) at 48 hpf. At 72 hpf, FS in Vinculin morphants was reduced to 42.97% ± 6.53% compared to control morphants (68.26% ± 1.5%), whereas ventricular chambers of Vinculin morphants became almost silent compared to controls at 96 hpf (MO-*vinculin*: 1.75% ± 4.95%; MO-control: 66.33% ± 3.87%).
(PDF)

S1 Movie Heart movie of a control-injected zebrafish embryo at 72 hpf. (MOV)

S2 Movie Heart movie of a MO1-*paxillin*-injected zebrafish embryo at 72 hpf. (MOV)

S3 Movie Heart movie of a MO1-*fak1a+fak1b* injected zebrafish embryo at 72 hpf. (MOV)

S1 Table Sequences of Morpholino antisense oligonucleotides. (PDF)

S2 Table Primer sequences. (PDF)
