# Supplementary material for: Paxillin and Focal Adhesion Kinase (FAK) Regulate Cardiac Contractility in the Zebrafish Heart
Source: PLoS One. 2016 Mar 8;11(3):e0150323. doi: 10.1371/journal.pone.0150323 (PMC4782988; doi:10.1371/journal.pone.0150323)
Supplement: S1 Fig — Amino acid sequence alignment of zebrafish (dr), mouse (mm) and human (hs) Paxillin, demonstrating high cross-species homology. Identical amino acids are shaded in black and shown as asterisks in the consensus line. Aminio acids with similar chemicals properties are shaded in gray. Highly homologous motifs such as the LD motifs and LIM domains are indicated. (PDF) [file pone.0150323.s001.pdf]

```
                      LD1
mmpxn        1 MDDLDALLADLESTTSHISKRPVFLSEEPPYSYPTGNHTYQEIAVPPPVPPPPSSEALNG
hspxn        1 MDDLDALLADLESTTSHISKRPVFLSEETPYSYPTGNHTYQEIAVPPPVPPPPSSEALNG
drpxn        1 MDDLDALLADLESTTSHISKQGVFLPEETPYSCPRGSDSYQDVSSPPRVISPP-AETLNG
consensus    1 ********************..***.**.***.*.*...**....**.*..**..*.***

mmpxn       61 TVLDPLDQWQPSGSRYAHQQPPSPLPVYSSSAKNSSASNTQDGVGSLCSRAGEEEHVYSF
hspxn       61 TILDPLDQWQPSSSRFIHQQPQSSSPVYGSSAKTSSVSNPQDSVGSPCSRVGEEEHVYSF
drpxn       60 SWVE-----KPESK-------HSSTQSFNSAPKSSSPRVSQS----------EEEHVYSF
consensus   61 .........*..... .... *..... *..*.** ...*. ... ... .********
                                                LD2
mmpxn      121 PNKQKSAEPSPTVMSSSLGSNLSELDRLLLELNAVCHSPPGFPADEAESSPPLPGALSPL
hspxn      121 PNKQKSAEPSPTVMSTSLGSNLSELDRLLLELNAVCHNPPGFPADEANSSPPLPGALSPL
drpxn       98 PNKQKSIESPTAVMNSSLGSNLSELDRLLLELNAVCHSTPSFPAEET--YPPKPASNTQR
consensus  121 ******.*....**..*********************..*.***.*.  ..**.*......
                                                              LD3
mmpxn      181 YGIPENNTPLGGKAGPLVKEKPKRNG-GRGLEDVRPSVESLLDELESSVPSPVPAITVNQ
hspxn      181 YGVPETNSPLGGKAGPLTKEKPKRNG-GRGLEDVRPSVESLLDELESSVPSPVPAITVNQ
drpxn      156 Y-VPENGVSSVVKAAPPKIEKPKRNIPAKVIEEVRPSVESLLNQLESSVPAAVPVSSVPM
consensus  181 *..**.......**.*. .******. ....*.*********..******..**...*..

mmpxn      240 G-EMSSPQRVTSSQQQTRISASSATRELDELMASLSDFK---------------------
hspxn      240 G-EMSSPQRVTSTQQQTRISASSATRELDELMASLSDFKIQGLEQRADGERCWAAGWPRD
drpxn      215 VSELRGVQEETPAQQQARISASSATRELDELMASLSDFKVQSN-----------------
consensus  241 . *....*..*..***.**********************..
                                                           LD4
mmpxn      278 -------------FMAQGKTGSSSPPGGLSKPGSQLDSMLGSLQSDLNKLGVATVAKGVC
hspxn      299 GGRSSPGGQDEGGFMAQGKTGSSSPPGGPPKPGSQLDSMLGSLQSDLNKLGVATVAKGVC
drpxn      258 -------------IMAQGKSSPTSVP----KQGNKLDNMLGSLQSDLNRLGVQTVAKGVC
consensus  301              .*****....*.*..  *.*..**.**********.***.*******
                                         LIM1
mmpxn      325 GACKKPIAGQVVTAMGKTWHPEHFVCTHCQEEIGSRNFFERDGQPYCEKDYHSLFSPRCY
hspxn      359 GACKKPIAGQVVTAMGKTWHPEHFVCTHCQEEIGSRNFFERDGQPYCEKDYHNLFSPRCY
drpxn      301 GACKKPIAGQVVTAMGRTWHPEHFVCTQCQEEIGSRNFFERDGQPYCEKDYHSLFSPRCY
consensus  361 ****************.**********.************************.*******
                                         LIM2
mmpxn      385 YCNGPILDKVVTALDRTWHPEHFFCAQCGAFFGPEGFHEKDGKAYCRKDYFDMFAPKCGG
hspxn      419 YCNGPILDKVVTALDRTWHPEHFFCAQCGAFFGPEGFHEKDGKAYCRKDYFDMFAPKCGG
drpxn      361 YCSGPILDKVVTALDKTWHPEHFFCAQCGSFFGPEGFHEKEGKAYCRKDYFDMFAPKCGG
consensus  421 **.*************.***************.**********.*******************
                                         LIM3
mmpxn      445 CARAILENYISALNTLWHPECFVCRECFTPFVNGSFFEHDGQPYCEVHYHERRGSLCSGC
hspxn      479 CARAILENYISALNTLWHPECFVCRECFTPFVNGSFFEHDGQPYCEVHYHERRGSLCSGC
drpxn      421 CARAILENYISALNSLWHPECFVCRECFTPFVNGSFFEHEGQPYCEAHYHERRGSLCSGC
consensus  481 **************.************************.******.*************
                                         LIM4
mmpxn      505 QKPITGRCITAMAKKFHPEHFVCAFCLKQLNKGTFKEQNDKPYCQSCFVKLFC
hspxn      539 QKPITGRCITAMAKKFHPEHFVCAFCLKQLNKGTFKEQNDKPYCQNCFLKLFC
drpxn      481 QKPITGRCITAMGKKFHPEHFVCAFCLKQLNKGTFKEQNDKPYCQSCFVKLFS
consensus  541 ************.********************************.**.***.
```

S1 Fig. Zebrafish Paxillin displays high amino acid sequence homology to murine and human Paxillin. Amino acid sequence alignment of zebrafish (dr), mouse (mm) and human (hs) Paxillin, demonstrating high cross-species homology. Identical amino acids are shaded in black and shown as asterisks in the consensus line. Aminio acids with similar chemicals properties are shaded in gray. Highly homologous motifs such as the LD motifs and LIM domains are indicated.
