# Supplementary material for: Paxillin and Focal Adhesion Kinase (FAK) Regulate Cardiac Contractility in the Zebrafish Heart
Source: PLoS One. 2016 Mar 8;11(3):e0150323. doi: 10.1371/journal.pone.0150323 (PMC4782988; doi:10.1371/journal.pone.0150323)
Supplement: S2 Fig — (A-C) Lateral view of MO1-paxillin (A) and MO1-paxillin+human paxillin mRNA (B) injected embryos at 72 hpf. (C) Bar graphs compare average of rescued embryos after co-injection of human paxillin mRNA compared to MO1-paxillin injected embryos (n = 3; *P = 0.0073). (D) Fractional shortening (FS) measurement of rescued embryos compared to Paxillin morphant embryos at 72 hpf (n = 5). (E, F) Lateral view of a (E) 5bp-mismatch MO (MO2-control) and (F) MO2-paxillin splice MO injected embryos at 72 hpf. The heart failure phenotype of MO2-paxillin splice morphants was identical to that of embryos injected with translation blocking paxillin MO (MO1-paxillin). (G) Bar graphs compare average of affected embryos after injection of MO2-paxillin compared to 5bp-mismatch MO (MO2-control) injected embryos at 72 hpf (n = 3; *P = 0.0001). (H) FS measurements of Paxillin morphant ventricles at 48, 72 and 96 hpf (n = 5 individuals per time point). (I) RT-PCR of control- and MO2-paxillin-injected embryos. Injection of MO2-paxillin caused partial and whole skipping of exon 2 (298 bp) leading to premature termination of Paxillin protein translation. Wild-type paxillin RNA was severely reduced in Paxillin morphants (527 bp product). (J) Western blot analysis of control- (MO1-control) and MO1-paxillin-injected embryos demonstrated high efficacy of MO-mediated paxillin gene knock-down. For each sample 50 embryos were pooled and 20 μg of protein lysate were loaded per lane. In vitro translated (IVT) zebrafish Paxillin protein was used as positive control. (PDF) [file pone.0150323.s002.pdf]

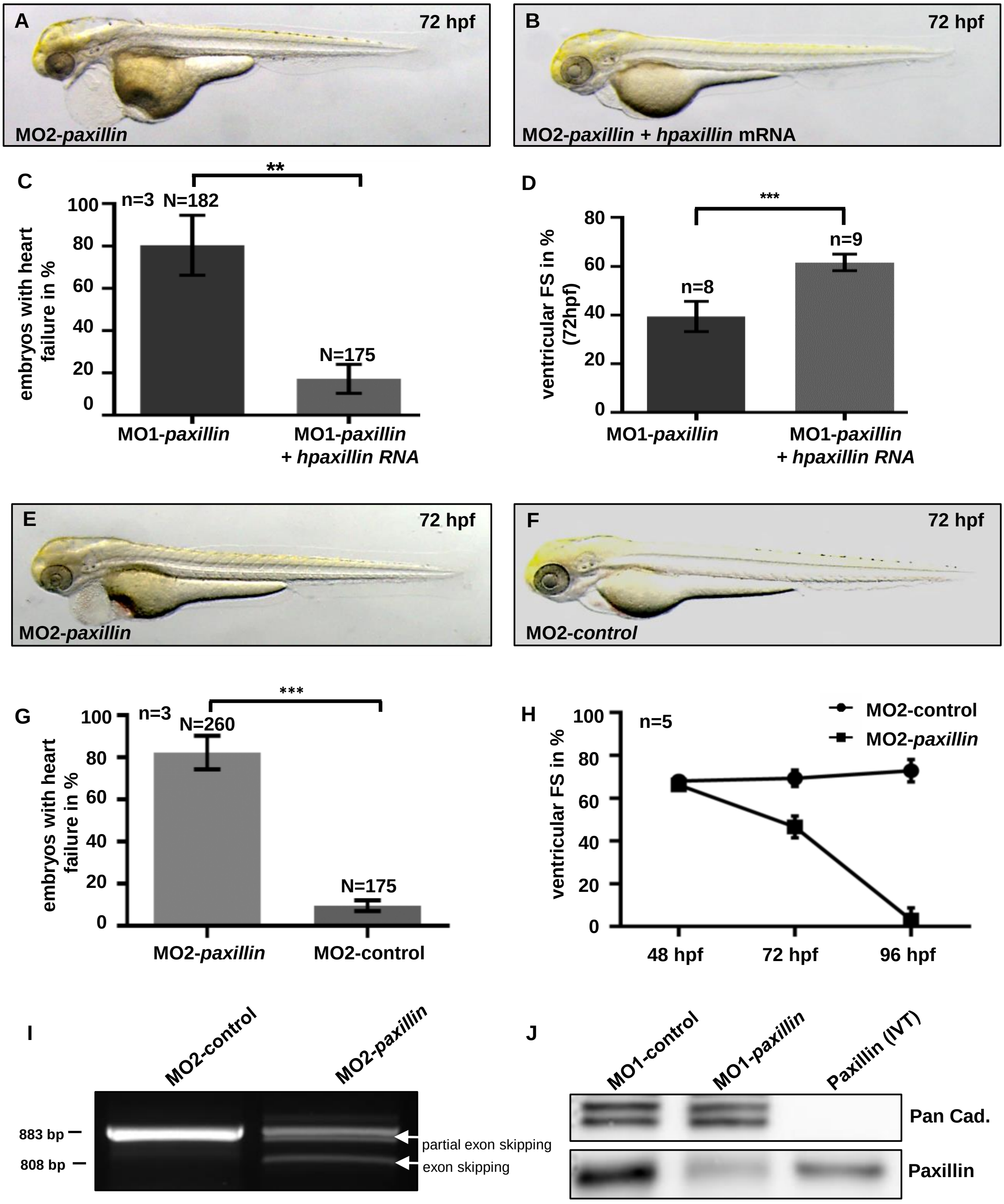

A
72 hpf
MO2-*paxillin*
B
72 hpf
MO2-*paxillin* + *hpaxillin* mRNA
C
**
n=3 N=182
N=175
embryos with heart failure in %
100
80
60
40
20
0
MO1-*paxillin*
MO1-*paxillin* + *hpaxillin RNA*
D
***
n=9
n=8
ventricular FS in % (72hpf)
80
60
40
20
0
MO1-*paxillin*
MO1-*paxillin* + *hpaxillin RNA*
E
72 hpf
MO2-*paxillin*
F
72 hpf
MO2-*control*
G
***
n=3
N=260
N=175
embryos with heart failure in %
100
80
60
40
20
0
MO2-*paxillin*
MO2-control
H
n=5
MO2-control
MO2-*paxillin*
ventricular FS in %
100
80
60
40
20
0
48 hpf
72 hpf
96 hpf
I
MO2-control
MO2-*paxillin*
883 bp
808 bp
partial exon skipping
exon skipping
J
MO1-control
MO1-*paxillin*
Paxillin (IVT)
Pan Cad.
Paxillin

S2 Fig. Injection of human *paxillin* mRNA rescues the cardiac phenotype of MO1-Paxillin morphants. (A-C) Lateral view of MO1-*paxillin* (A) and MO1-*paxillin*+human *paxillin* mRNA (B) injected embryos at 72 hpf. (C) Bar graphs compare average of rescued embryos after co-injection of human *paxillin* mRNA compared to MO1-*paxillin* injected embryos (n=3; **P*=0.0073). **(D)** Fractional shortening (FS) measurement of rescued embryos compared to Paxillin morphant embryos at 72 hpf (n=5). **(E, F)** Lateral view of a (E) 5bp-mismatch MO (MO2-control) and (F) MO2-*paxillin* splice MO injected embryos at 72 hpf. The heart failure phenotype of MO2-*paxillin* splice morphants was identical to that of embryos injected with translation blocking *paxillin* MO (MO1-*paxillin*). **(G)** Bar graphs compare average of affected embryos after injection of MO2-*paxillin* compared to 5bp-mismatch MO (MO2-control) injected embryos at 72 hpf (n=3; **P*=0.0001). **(H)** FS measurements of Paxillin morphant ventricles at 48, 72 and 96 hpf (n=5 individuals per time point). **(I)** RT-PCR of control- and MO2-*paxillin*-injected embryos. Injection of MO2-*paxillin* caused partial and whole skipping of exon 2 (298 bp) leading to premature termination of Paxillin protein translation. Wild-type *paxillin* RNA was severely reduced in Paxillin morphants (527 bp product). **(J)** Western blot analysis of control- (MO1-control) and MO1-*paxillin*-injected embryos demonstrated high efficacy of MO-mediated *paxillin* gene knock-down. For each sample 50 embryos were pooled and 20 µg of protein lysate were loaded per lane. *In vitro* translated (IVT) zebrafish Paxillin protein was used as positive control.
