# Supplementary material for: Paxillin and Focal Adhesion Kinase (FAK) Regulate Cardiac Contractility in the Zebrafish Heart
Source: PLoS One. 2016 Mar 8;11(3):e0150323. doi: 10.1371/journal.pone.0150323 (PMC4782988; doi:10.1371/journal.pone.0150323)
Supplement: S3 Fig — (A, B) Lateral view of (A) control MO (MO2-control) and (B) MO2-fak1a/fak1b-injected embryos at 72 hpf. The heart failure phenotype of fak1a/fak1b splice morphants was identical to that of embryos injected with the translation blocking FAK MOs (MO1-fak1a/fak1b). (C) RT-PCR of control-, MO2-fak1a- and MO2-fak1b-injected embryos. Injection of MO2-fak1a and MO2-fak1b caused intron integration (808 bp MO2-fak1a; 883 bp MO2-fak1b) leading to premature termination of FAK1a and FAK1b protein translation, respectively. Wild-type fak1a and fak1b RNA was severely reduced in the respective morphants (150 bp MO2-fak1a; 474 bp MO2-fak1b). (D) Western Blot analysis of control and fak1a/fak1b morphant embryos with an antibody against FAK. For each sample 50 embryos were pooled and 20 μg of protein lysate were loaded per lane. (PDF) [file pone.0150323.s003.pdf]

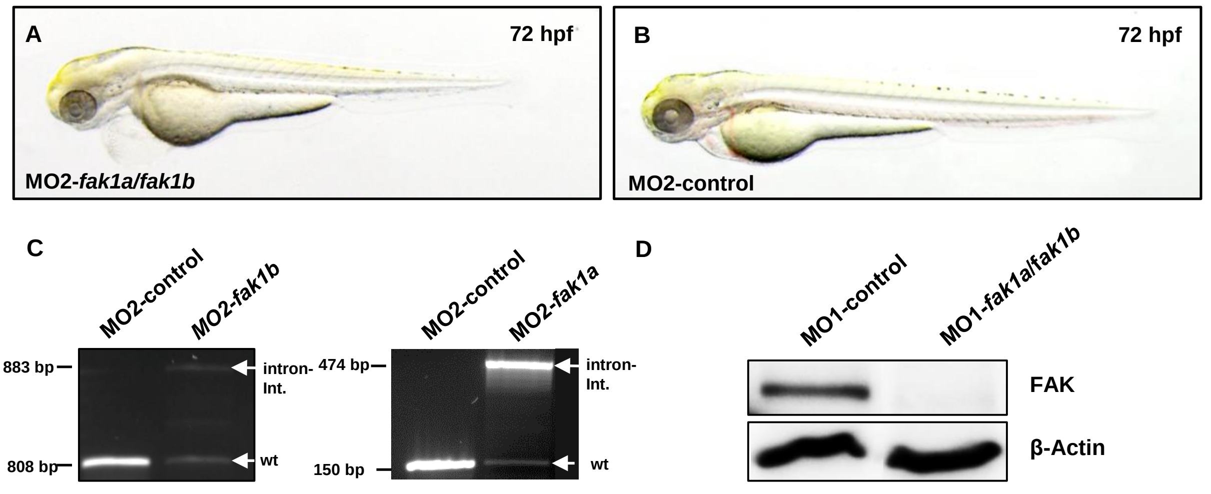

S3 Fig. Co-injection of MO2-*fak1a* and MO2-*fak1b* results in defective splicing and heart failure *in vivo.* (A, B) Lateral view of (A) control MO (MO2-*control)* and (B) MO2-*fak1a/fak1b*-injected embryos at 72 hpf. The heart failure phenotype of *fak1a*/*fak1b* splice morphants was identical to that of embryos injected with the translation blocking FAK MOs (MO1-*fak1a/fak1b)*. **(C)** RT-PCR of control-, MO2-*fak1a-* and MO2-*fak1b*-injected embryos. Injection of MO2-*fak1a* and MO2-*fak1b* caused intron integration (808 bp MO2-*fak1a*; 883 bp MO2-*fak1b*) leading to premature termination of FAK1a and FAK1b protein translation, respectively. Wild-type *fak1a* and *fak1b* RNA was severely reduced in the respective morphants (150 bp MO2-*fak1a*; 474 bp MO2-*fak1b*). **(D)** Western Blot analysis of control and fak1a/fak1b morphant embryos with an antibody against FAK. For each sample 50 embryos were pooled and 20 µg of protein lysate were loaded per lane.
