# Supplementary material for: Paxillin and Focal Adhesion Kinase (FAK) Regulate Cardiac Contractility in the Zebrafish Heart
Source: PLoS One. 2016 Mar 8;11(3):e0150323. doi: 10.1371/journal.pone.0150323 (PMC4782988; doi:10.1371/journal.pone.0150323)
Supplement: S4 Fig — (A, B) Lateral view of MO-vinculin (A) and vinculin 5bp-mismatch-MO (MO-control) (B) injected embryos at 72 hpf. (C) Bar graphs compare average of affected embryos after MO injection (MO-vinculin 82.85% ± 6.95%; MO-control 7.3% ± 3.76%; *P<0.0001). (D) Fractional shortening (FS) measurements of Vinculin morphant ventricles compared to control injected embryos at 48, 72 and 96 hpf. FS of Vinculin morphant ventricles was not affected compared to corresponding 5bp-mismatch-MO injected embryos (MO-vinculin 68.01% ± 3.52% vs. MO-control: 66.08% ± 3.12%) at 48 hpf. At 72 hpf, FS in Vinculin morphants was reduced to 42.97% ± 6.53% compared to control morphants (68.26% ± 1.5%), whereas ventricular chambers of Vinculin morphants became almost silent compared to controls at 96 hpf (MO-vinculin: 1.75% ± 4.95%; MO-control: 66.33% ± 3.87%). (PDF) [file pone.0150323.s004.pdf]

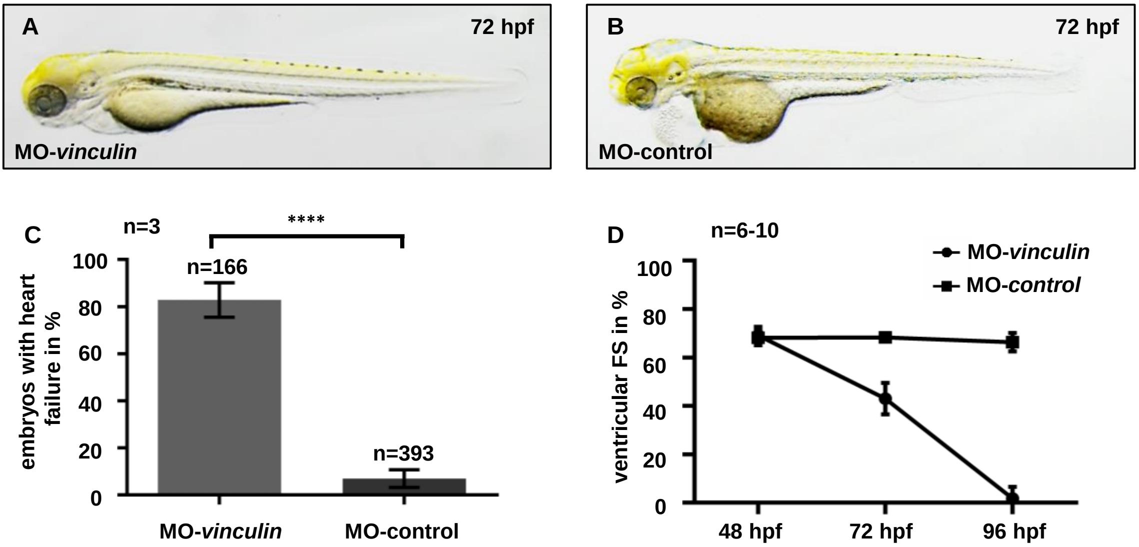

S4 Fig. Knockdown of Vinculin results in cardiac contractile dysfunction. (**A, B**) Lateral view of MO-*vinculin* (A) and *vinculin* 5bp-mismatch-MO (MO-control) (B) injected embryos at 72 hpf. **(C)** Bar graphs compare average of affected embryos after MO injection (MO-*vinculin* 82.85% ± 6.95%; MO-control 7.3% ± 3.76%; **P*<0.0001). **(D)** Fractional shortening (FS) measurements of Vinculin morphant ventricles compared to control injected embryos at 48, 72 and 96 hpf. FS of Vinculin morphant ventricles was not affected compared to corresponding 5bp-mismatch-MO injected embryos (MO-*vinculin* 68.01% ± 3.52% vs. MO-control: 66.08% ± 3.12%) at 48 hpf. At 72 hpf, FS in Vinculin morphants was reduced to 42.97% ± 6.53% compared to control morphants (68.26% ± 1.5%), whereas ventricular chambers of Vinculin morphants became almost silent compared to controls at 96 hpf (MO-*vinculin*: 1.75% ± 4.95%; MO-control: 66.33% ± 3.87%).
