# Supplementary material for: Paxillin and Focal Adhesion Kinase (FAK) Regulate Cardiac Contractility in the Zebrafish Heart
Source: PLoS One. 2016 Mar 8;11(3):e0150323. doi: 10.1371/journal.pone.0150323 (PMC4782988; doi:10.1371/journal.pone.0150323)
Supplement: S1 Table — (PDF) [file pone.0150323.s008.pdf]

## S1 Table. Sequences of Morpholino antisense oligonucleotides

| Gene name | Accession # | MO target | MO sequence 5'-3' | MO conc. |
|---|---|---|---|---|
| *paxillin (pxn)* | NM_201588.1 | ATG | CGAGAAGAGCATCTAAATCGTCCAT | 5.4 ng |
| | | E2I2 | TTGGTGCTTTACAAACTGACCTTCA | 4.5 ng |
| | | ATG mismatch | CCAGAACAGCATATAAATCCTCAAT | 5.4 ng |
| | | splice mismatch | TTCGTCCTTTAAAAACTGACATTAA | 4.5 ng |
| *focal adhesion kinase 1a (fak1a)* | XM_009292415.1 | ATG | GGGTCCAGGTAAGCCGCTGCCATG | 2.7 ng |
| | | E5I5 | CCAGATTAAACTATCCATCACCTGA | 2.7ng |
| | | ATG mismatch | GCGTCCACGTAACCCGCTCCAATG | 2.7 ng |
| | | splice mismatch | CCAGATTAAACTATCCATCACCTGA | 2.7 ng |
| *focal adhesion kinase 1b (fak1b)* | AY196213.1 | ATG | TGGGCTCCAGGAATGCCGTCGCCAT | 2.15 ng |
| | | I5E6 | ACACACTCAGAGACACTCACCTGCT | 2.2 ng |
| | | ATG mismatch | TCGGCTCAAGGAATCCCGTCCCAAT | 2.15 ng |
| | | splice mismatch | ACACACTCAGAGACACTCACCTGCT | 2.2 ng |
| *vinculin* | NM_001128681.1 | ATG | GTCTTGGTATGGAAAACTGGCATCC | 5.3 ng |
| | | ATG mismatch | GTATTGCTATGGAAAAATGGAATAC | 5.3 ng |
| *human beta-globin intron mutation* | | standard control | CCTCTTACCTCAGTTACAATTTATA | adjusted to the respective MO |
