# Supplementary material for: Paxillin and Focal Adhesion Kinase (FAK) Regulate Cardiac Contractility in the Zebrafish Heart
Source: PLoS One. 2016 Mar 8;11(3):e0150323. doi: 10.1371/journal.pone.0150323 (PMC4782988; doi:10.1371/journal.pone.0150323)
Supplement: S2 Table — (PDF) [file pone.0150323.s009.pdf]

S2 Table: **Primer sequences**

primer name	Sequence 5‘-3‘
*paxillin*_splice-assay_fw	ACCTCCCACATTTCCAAACA
*paxillin*_splice-assay_rv	TTGCAGGTATATTGCGCTTG
*fak1a*_splice-assay_fw	CTGCGCTTGAGTCATCTGAG
*fak1a*_splice-assay_rv	CAACTTGATCTGCAATCTCCA
*fak1b*_splice-assay_fw	AGTGCAGCATGTGGTGTGTT
*fak1b*_splice-assay_rv	AGGTTGGCGAACTGCTTAAA
*rpl13*_qPCR_*fw*	TCTGGAGGACTGTAAGAGGTATGC
*rpl13*_qPCR_rv	AGACGCACAATCTTGAGAGCAG
β-actin2 qPCR_fw	GCAGAAGGAGATCACATCCCTGGC
β-actin2 qPCR_rv	CATTGCCGTCACCTTCACCGTTC
*paxillin*_qPCR_fw	CGCCGTCATGAACTCCTC
*paxillin*_qPCR_rv	GTTTGGGTGGGTAGGTCTCTT
*vinculin*_qPCR_fw	TGACATTCTTCGTTCCATCG
*vinculin*_qPCR_rv	GCCTCTGGAGTATCGCCTTT
fak1a qPCR_fw	GCGCTTGAGTCATCTGAGAA
fak1a qPCR_rv	GTATCGAATCCGTAGCTCATATCTC
fak1b qPCR_fw	TGCTGGACAACATAATCACTATCA
fak1b qPCR_rv	TCGAGGATGGACGTCTGC
